# Cytocompatibility of 3D printed dental materials for temporary restorations on fibroblasts

**DOI:** 10.1186/s12903-020-01150-2

**Published:** 2020-06-01

**Authors:** Jung-Hyun Park, Hyun Lee, Jong-Woo Kim, Ji-Hwan Kim

**Affiliations:** 1 Department of Dental Laboratory Science and Engineering, Hana Sciences Hall B #374, Korea University, 145, Anam-ro, Seongbuk-gu, Seoul, Republic of Korea 02841; 2 Institute of Health Science Research, Hana Sciences Hall B #473, Korea University, 145, Anam-ro, Seongbuk-gu, Seoul, Republic of Korea 02841; 3 School of Biomedical Engineering, Hana Sciences Hall B #473, Korea University, 145, Anam-ro, Seongbuk-gu, Seoul, Republic of Korea 02841

**Keywords:** 3D printing, Dental resin, Temporary, Fibroblasts, Cell adhesion, Cell proliferation

## Abstract

**Background:**

Three-dimensional (3D) printing is widely used in the fabrication of dental prostheses; however, the influence of dental materials used for 3D printing on temporary restoration of fibroblasts in tissues is unclear. Thus, the influence of different dental materials on fibroblasts were investigated.

**Methods:**

Digital light processing (DLP) type 3D printing was used. Specimens in the control group were fabricated by mixing liquid and powder self-curing resin restoration materials. The temporary resin materials used were Model, Castable, Clear-SG, Tray, and Temporary, and the self-curing resin materials used were Lang dental, Alike, Milky blue, TOKVSO CUREFAST, and UniFast III. Fibroblast cells were cultured on each specimen and subsequently post-treated for analysis. Morphology of the adhered cells were observed using a confocal laser scanning microscope (CLSM) and a scanning electron microscope (SEM).

**Results:**

CLSM and SEM cell imaging revealed that the 3D printed material group presented better cell adhesion with well-distributed filopodia compared to that in the conventional resin material group. Cell proliferation was significantly higher in the 3D printing materials.

**Conclusion:**

Superior cytocompatibility of the specimens fabricated through 3D printing and polishing process was demonstrated with the proof of better cell adhesion and higher cell proliferation.

## Background

Temporary restoration materials are widely used in dental clinics and are important for predicting the successful prognosis of endodontic treatment including inlay, onlay, crown, and bridge [1, 2]. These materials are also used to protect the invasion of external substances and microorganism and to help the recovery of tooth functions, including mastication and esthetics [3, 4].

Recently, the introduction of three-dimensional (3D) printing equipment has enabled quick fabrication of dental restoration through the use of an automatized protocol [5]. Unlike conventional fabrication methods of temporary restorations, such as resin curing or CAD/CAM milling, digital dentistry is dominantly driven by 3D printing technology [6, 7].

The resin curing method, which is the conventional fabrication method of temporary restorations, adopts the curing reaction of an acrylic resin system, resulting from the reaction of dibutyl phthalate, a plasticizer, due to the interaction between the powder and liquid when mixed. The powder contains poly-methyl methacrylate (PMMA), a reaction initiator, and the liquid contains methyl methacrylate (MMA) and a small amount of inhibitor [8, 9]. This manually driven technique has advantages including the ability to create the desired shape, quick hardening, and excellent handling. However, the conditions of the work environment are strict and the process is, thus, time consuming.

Three-dimensional (3D) printing fabrication is classified into several subtypes, including extrusion, wire, granular, and light polymerized, based on the type of the technology used. Temporary restorative resin materials for dental use are treated with the use of digital light processing (DLP) technology, which adopts light polymerized technique to enable the processing of polymers [10, 11]. This technology prints the resins layer by layer as it hardens, by projecting light in the desired shape for photo hardening liquid resins [12]. This technology is advantageous, as it is capable of printing without the use of any supporting beam inside the sculpture, and it produces printing products with excellent details and smooth surfaces and has a high printing speed [13]. On the other hand, there are some limitations, such as the colors of applicable materials are limited and the base materials and printer itself are expensive [14]. In addition, the more exquisite the printed product is, the more complicated is the work in creating the printed product [15].

Temporary restorations that are fabricated through such method undergo polishing and cleansing processes and are used until the placement of the permanent restoration for recovery of the function of the lost teeth [16]. Since temporary restorations are placed inside the mouth, temporary restorative resin materials are used based on analyses of their material properties [17]. There is a lack of studies that assess “what kinds of relationships exist between the negative micro influences of the restorations on intraoral living tissues and the restorations applied after the secondary processing following fabrication using such a method.”

According to a previous study, the monomers leached from the temporary restorative resin materials may cause dental pulp injury, oral mucosal irritation, and allergic reaction [18]. when it comes to using dental polymers, an important issue is that these influence the survival and physiological activities of cells, and monomer diffusion influences the viability of gingival cells and physiological activities of dental pulps [19]. Penetrating into pulp tissues, resin brings about cytotoxicity and genetic damage [20]. Also, poly-acrylic resin causes gingivitis and periodontitis by discharging cytokine and chemokine, which are inflammatory protein syntheses in fibroblast [21]. Furthermore, these may also cause hypersensitive asthmatic response, conjunctivitis, neurologic response, and epithelial response in dentists and other dental clinic staff [22]. Toxicity of acrylic resins has been reported from in-vitro cell experiments [23].

**Fig. 1:**
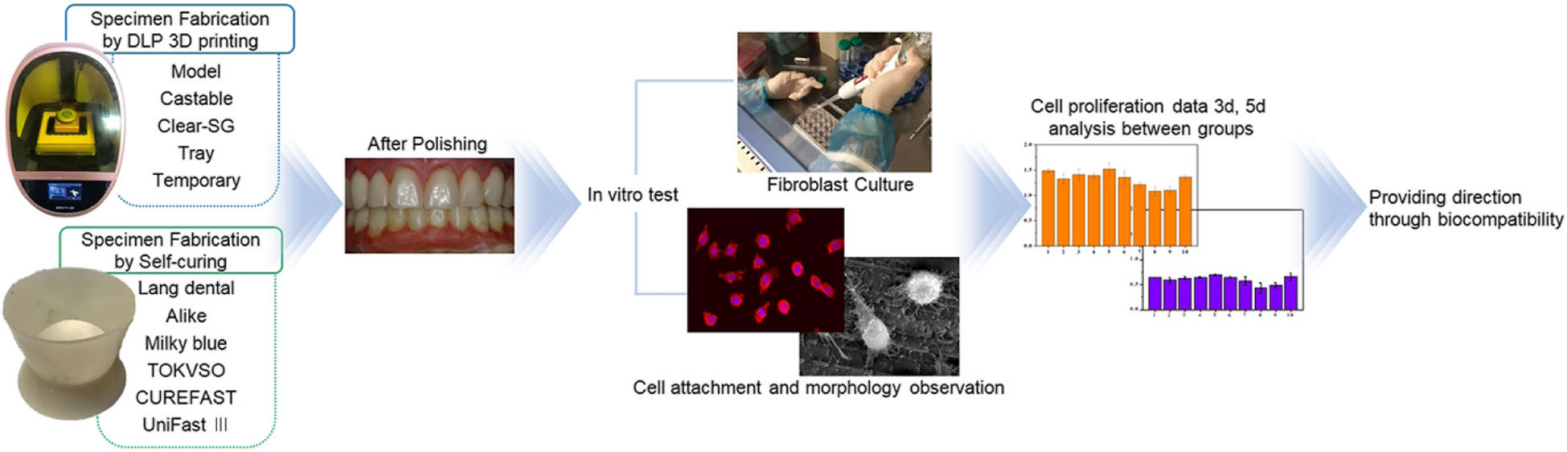
Dental fabrication process of temporary restorations by DLP 3D printing and Self-curing method cytocompatibility of the fibroblast cell

Hence, the biocompatibility of all dental resin materials used in temporary restoratives that are to be placed in the mouth must be thoroughly studied before and after their clinical use. This study aims to investigate the influence of dental materials fabricated either by 3D printing technology or self-curing technology on fibroblasts. We also assessed the cytocompatibility of dental materials used for temporary restoratives by analyzing and comparing cell adhesion and cell proliferation (Fig. 1). A null hypothesis of this study is that there are no statistically significant differences in fibroblast cytocompatibility between 3D printing technology-based and existing self-curing technology-based temporary restorative resin materials.

## Methods

### Preparation of specimen

**Fig. 2:**
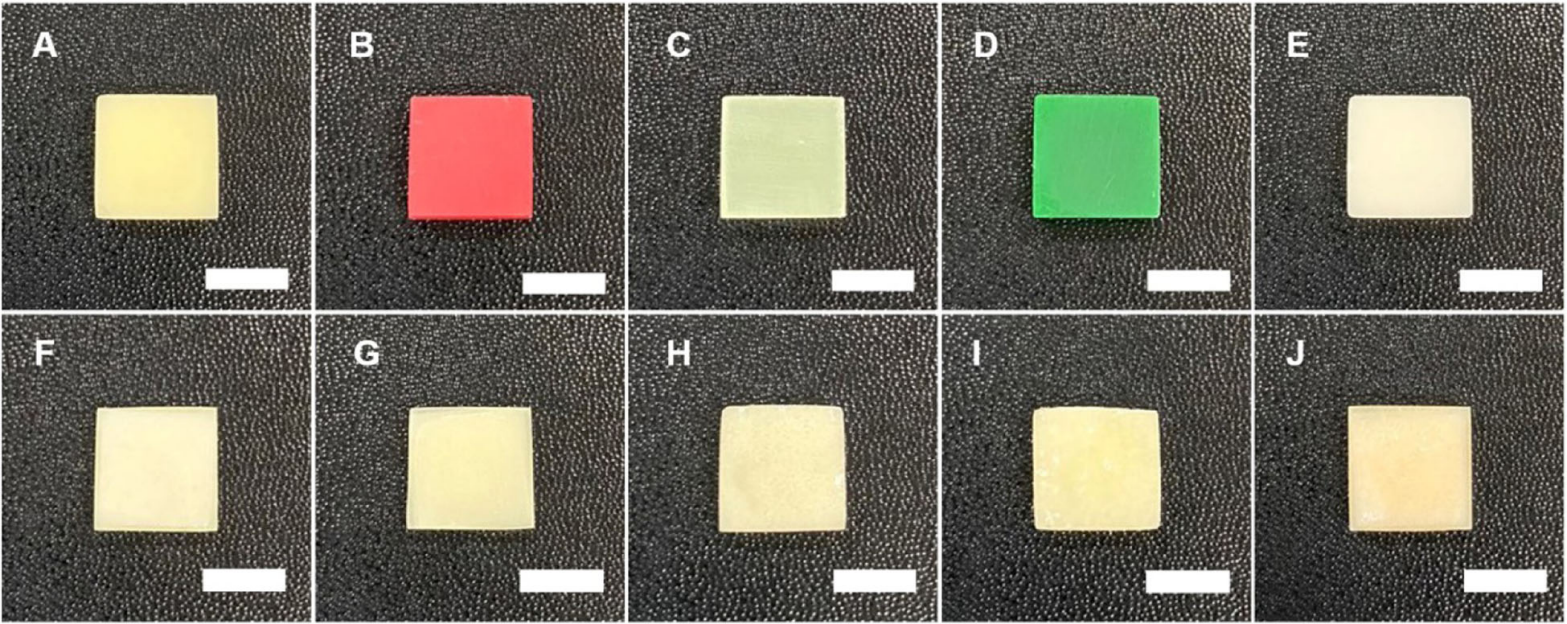
Specimens of temporary restoration resin material produced by DLP 3D printing method and self-curing method to culture fibroblasts (scale bar: 10mm). **a**: Model. **b**: Castable. **c**: Clear-SG. **d**: Tray. **e:** Temporary. **f**: Lang dental. **g:** Alike. **h**: Milky blue. **i**: Tokuso Curefast. **j**: Unifast III

The ten specimens were designed in sizes of 10 X 10 X 3 mm, a size capable of use in cell culture (Fig. 2).For this study, a DLP 3D print was designed using a CAD design program (Exocad; GmbH, Darmstadt, Germany) and then this was converted to a stl file using DLP 3D modeling software (Zenith D ZD200 V1.0.5). UV curing was applied to a temporary restorative resin material with a layer thickness of 120 μm, printing speed 20 min and a light source of 405 μm LED using a 3D printer (Zenith D; Dentis, South Korea) and this was printed out to manufacture a specimen. Dental materials that are commercially used to fabricate temporary restorations were used and the materials were MODEL (ZMD-1000B MODEL; Dentis, South Korea), CASTABLE (ZMD-1000B CASTABLE; Dentis, South Korea), CLEAR-SG (ZMD-1000B CLEAR-SG; Dentis, South Korea), TRAY (ZMD-1000B TRAY; Dentis, South Korea), and TEMPORARY (ZMD-1000B TEMPORARY; Dentis, South Korea). Existing temporary resin materials fabricated in self-curing method used were Jet Tooth Shade Acrylic Resin (Lang REF 1430; Lang Dental Mfg, USA), Alike (ALIKE 81; GC America, USA), Milky blue (MILKY BLUE; Nissin Dental, Japan), Tokuso Curefast (CUREGRACE; Tokuyama Dental, Japan), and Uni-Fast III (A3; GC Dental Product co, Japan).

In this study, Self-curing resin specimens for each material were fabricated and 2 g of the powder of the standard mixing ratio and approximately 1 ml of the liquid that is exclusive for each resin powder were used for the self-curing resin materials. After pouring the liquid into a rubber cup, the powder was added and mixed with a spatula for approximately 10 s. The specimens were fabricated when the mixture adopted a dough state, which is free of adhesiveness and allows easy shaping. After the final setting, fabrication of all specimens were completed using the polishing process standard in dental restoration fabrication.

The specimens were polished using a carbide bur, silicone point, and finishing wheel and cleansed. Polishing procedure for cell culture was conducted for both of study groups using SiC paper to eliminate the effect of roughness which was generated during fabrication.

### In-vitro cell culture

A fibroblast cell line (L929; derivative of strain L, *Mus musculus* mouse, ATCC, CRL-2593) was used in this experiment. Fibroblast cells were cultured at 37 °C in a humidified incubator containing 5% CO_2_. The culture medium used was minimum essential medium (α-MEM; Welgene Co., Ltd., Seoul, Korea) containing 10% fetal bovine serum (FBS), 1% penicillin streptomycin, 10 mM β-glycerophosphate (Sigma), and 10 μg/mL ascorbic acid. Cell culture maintenance was performed by washing the cells with Dulbecco’s phosphate-buffered saline (DPBS) followed by cell detachment using trypsin-EDTA. The detached cells were then suspended in culture medium, centrifuged, counted using trypan blue dye, plated in culture plates (10 mL, 3 × 10^4^ cells/mL), and cultured at 37 °C.

### Cell attachment analysis

The surface and edge of the 10 specimens made in each of the 10 materials were trimmed in the shape of a plate of a size sufficient for cell culture. Cell morphology was compared using confocal laser scanning microscopy (CLSM; C1 Plus, Inverted IX81, Olympus, Japan) and scanning electron microscopy (SEM; JSM-6360; JEOL Techniques, Tokyo, Japan). Fibroblast cells with a density of 3 × 10^4^ cells/mL were cultured for 1 day on each of the specimens sterilized with 70% ethanol. To prepare for CLSM observation, cells were then fixed with 4% paraformaldehyde for 10 min, permeabilized with 0.1% Triton X, and blocked with 1% bovine serum albumin. The specimens were then immersed in phalloidin and 4′,6-diamidino-2-phenylindole to stain the cellular actin and nuclei, respectively. Prior to SEM characterization, attached cells were rinsed with DPBS and fixed with 2.5% glutaraldehyde for 10 min. Thereafter, sequential dehydration was conducted by 5 min immersions in 75, 95, and 100% ethanol, and the specimens were treated with 1,1,1,3,3,3-hexamethyldisilazane for 10 min.

### Analysis of cell proliferation

For this experiment, cell proliferation was attempted and observed from the specimens fabricated using the 3D printing method and resin specimens fabricated using the self-curing method. The plates were then placed into the wells and fibroblast cells were cultured. After 3 and 5 days of culturing, the specimens were rinsed with DPBS. For the methoxyphenyl tetrazolium salt (MTS) assay, FBS-absent medium containing 10% of MTS was added to each of the specimens and incubated at 37 °C for 2 h. 200 μl of the medium was then placed into a 96-well plate and absorbance was measured at 490 nm using a Micro-reader (Model 550; BioRad, USA).

### Statistical analysis

Tests for normality were performed using a Kolmogorov-Smirnov test and Shapiro-Wilk test. Levene’s test was performed to test homogeneity of variances. After performing Kruskal-Wallis test using non-parametric statistics and statistically significant differences were presented. Pair-wise comparison was performed, and inter-group comparison was done within the confidence interval of 95%. Statistical significance was indicated as *p < .05 and **p < .01, the sample size (*n* = 3). Statistical analyses were performed using IBM SPSS (IBM SPSS 25.0 Inc., Chicago, IL, USA).

## Results

### Cell attachment analysis

**Fig. 3:**
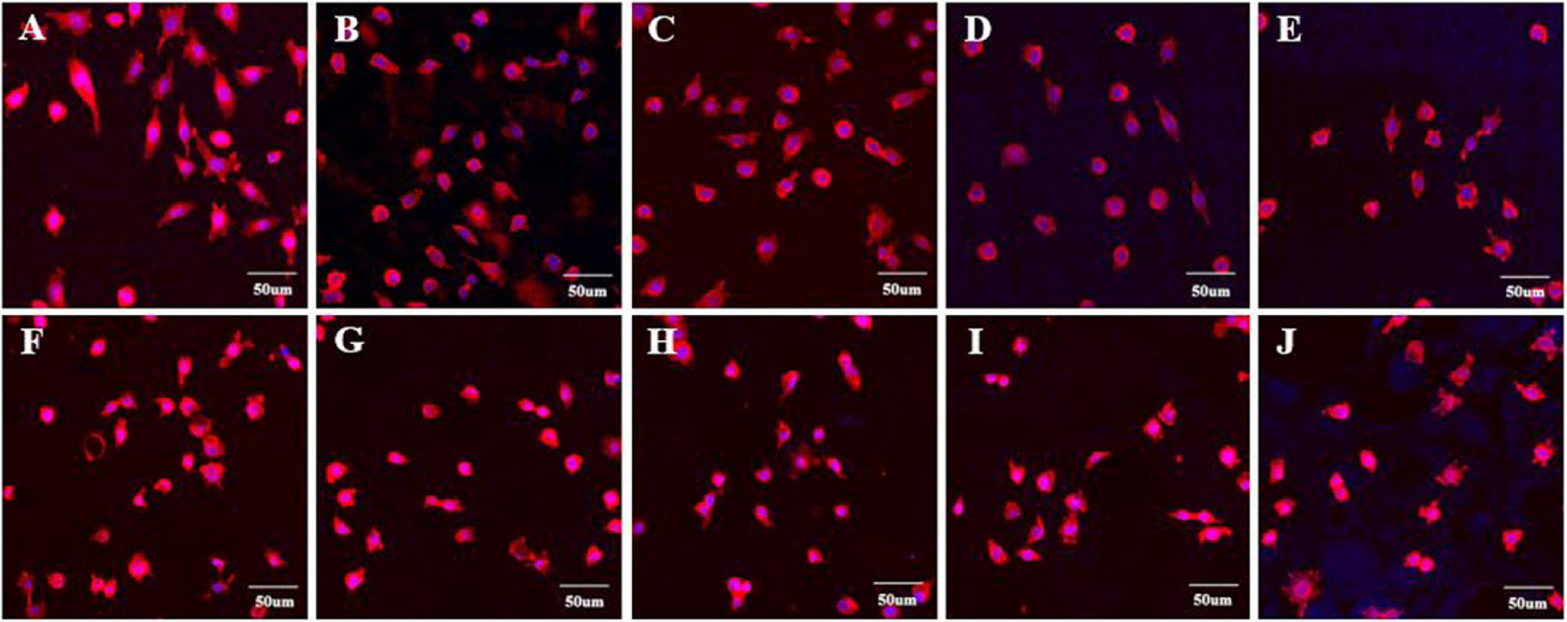
CLSM measurement of specimens made with DLP 3D printing and self-curing resin after 24 h fibroblast culture (3 × 10^4^ cells/mL). **a**: Model. **b**: Castable. **c**: Clear-SG. **d**: Tray. **e**: Temporary. **f**: Lang dental. **g:** Alike. **h**: Milky blue. **i**: Tokuso Curefast. **j**: Unifast III

**Fig. 4:**
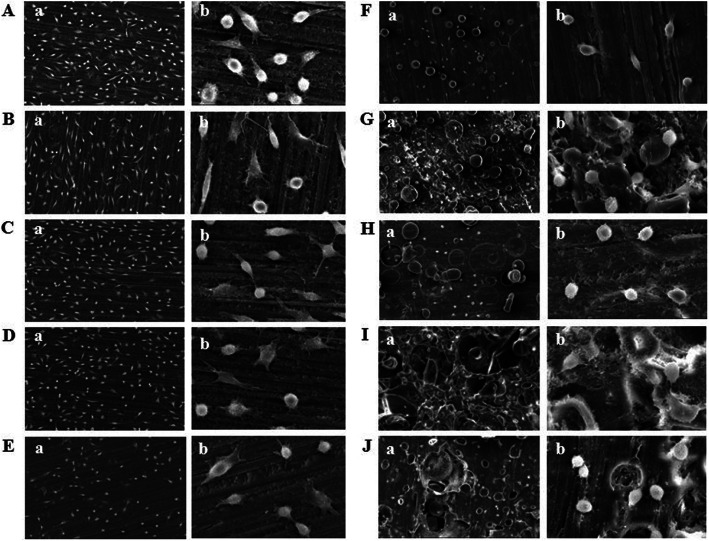
Results of SEM of Cell measurements of DLP 3D printing and self-curing resin specimens after 24 h fibroblast cell culture (3 × 10^4^ cells/mL). **a**: Model. **b**: Castable. **c**: Clear-SG. **d**: Tray. **e**: Temporary. **f**: Lang dental. **g**: Alike. **h**: Milky blue. **i**: Tokuso Curefast. **j**: Unifast III

The morphology and adhesion of fibroblasts in this in-vitro cell experiment using the specimens fabricated by 3D printing and self-curing resin technology are presented in Figs. 1, 2. A relatively high number of multi-nucleate cells, dyed in blue, and well-stretched cytoplasm, dyed in red, adhering on the specimens fabricated with 3D printing were observed using CLSM (Fig. 3a-e). SEM observation of cells presented well-stretched cytoplasm of fibroblasts and a greater number of cells adhering to the 3D-printed specimens (Fig. 4a-e). On the other hand, relatively fewer adhered cells and poor stretch of cell filopodia were observed on the specimens fabricated by using self-curing resins, due to their porous surface (Fig. 4f-j).

### Analysis of cell proliferation

**Fig. 5:**
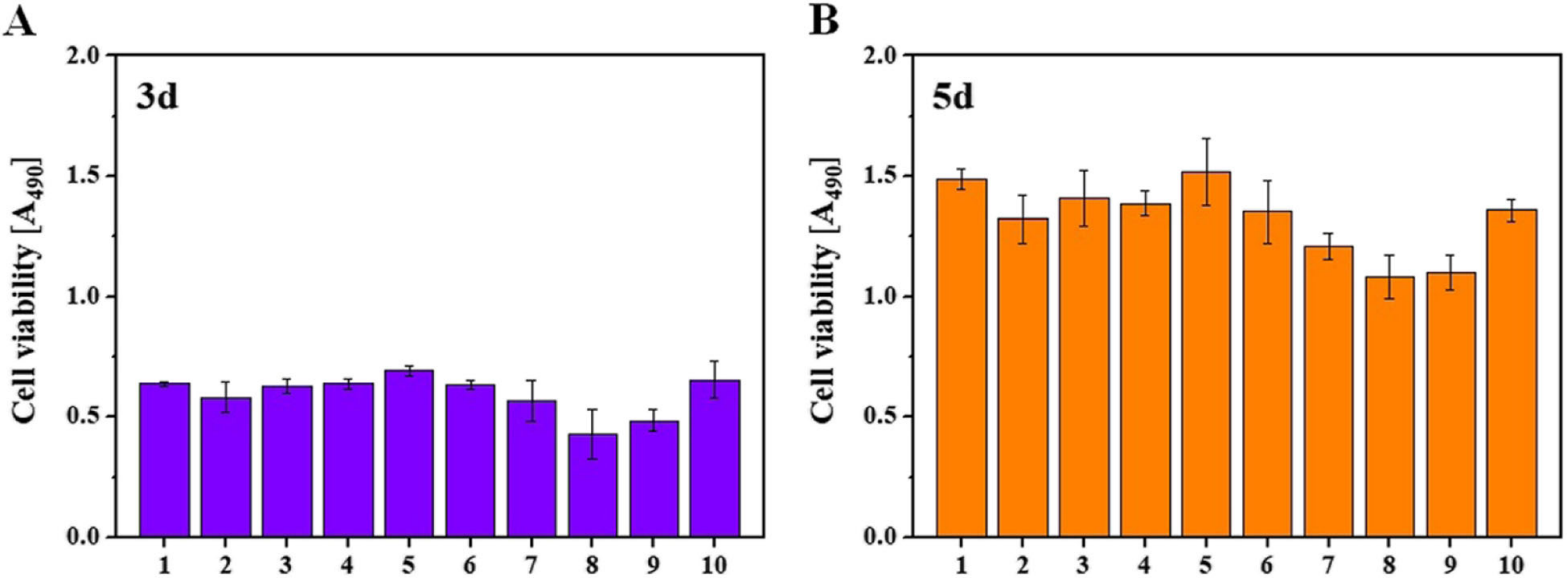
Fibroblasts MTS assay results measured after 3 and 5 days of cell proliferation. **a**: 3 days. **b**: 5 days. 1: Model. 2: Castable. 3: Clear-SG. 4: Tray. 5: Temporary. 6: Lang dental. 7: Alike. 8: Milky blue. 9: Tokuso Curefast. 10: Unifast III

Cell proliferation of fibroblasts, an in-vitro experiment, was measured 3 days and 5 days after the cell spreading through MTS assay (Fig. 5). The results of the measurement were statistically presented by stating the inter-group difference of light absorbance between 3D-printed specimen group and self-cured specimen group within confidence interval of 95% and level of significance as 0.05 (Tables 1, 2, 3). From the measurement taken after 3 days, among the self-curing resin materials, Milky blue presented average and standard deviation as 0.429 ± 0.10, which is statistically significantly small value; however, Uni-Fast III presented average and standard deviation as 0.655 ± 0.07, which is statistically significantly large value (Table 1). Among 3D printing resin materials, Castable presented average and standard deviation as 0.58 ± 0.06, which is statistically significantly small value; however, Temporary presented average and standard deviation as 0.69 ± 0.01, which is statistically significantly large value (Table 1). From the measurement after 5 days, among the self-curing resin materials, Milky blue presented an average and standard deviation as 1.08 ± 0.09, which is statistically significantly small value; however, Uni-Fast III presented average and standard deviation as 1.35 ± 0.04, which is statistically significantly large value (Table 2). Among 3D printing resin materials, Castable presented average and standard deviation as 1.32 ± 0.10, which is statistically significantly small value; however, Temporary presented average and standard deviation as 1.51 ± 0.13, which is statistically significantly large value (Table 2). Overall, a greater number of cells were found on the 3D printing resin materials compared to the self-curing resin materials.

**Table 1:** Statistical results of MTS assay measured after 3 days fibroblast proliferation

Materials	Absorbance			Mean	±	SD	Median	C.I
Model	0.637	0.648	0.626	0.637	±	0.011	0.637	0.625–0.649
Castable	0.597	0.512	0.638	0.582	±	0.064	0.597	0.510–0.654
Clear-SG	0.662	0.615	0.608	0.628	±	0.029	0.615	0.595–0.661
Tray	0.659	0.634	0.618	0.637	±	0.021	0.634	0.613–0.661
Temporary	0.702	0.671	0.703	0.692	±	0.018	0.702	0.672–0.712
Lang dental	0.658	0.622	0.627	0.636	±	0.020	0.627	0.613–0.659
Alike	0.525	0.516	0.668	0.570	±	0.085	0.525	0.474–0.666
Milky blue	0.437	0.527	0.324	0.429	±	0.102	0.437	0.314–0.544
Tokuso Curefast	0.518	0.509	0.431	0.486	±	0.048	0.509	0.432–0.540
Unifast III	0.721	0.572	0.674	0.656	±	0.076	0.674	0.570–0.742

**Table 2:** After 5 days of fibroblast cell proliferation, the statistical results of MTS assay

Materials	Absorbance			Mean	±	SD	Median	C.I
Model	1.535	1.463	1.465	1.488	±	0.041	1.465	1.442–1.534
Castable	1.311	1.228	1.43	1.323	±	0.102	1.311	1.208–1.438
Clear-SG	1.383	1.308	1.533	1.408	±	0.115	1.383	1.278–1.538
Tray	1.403	1.328	1.428	1.386	±	0.052	1.403	1.327–1.445
Temporary	1.661	1.388	1.505	1.518	±	0.137	1.505	1.363–1.673
Lang dental	1.219	1.365	1.474	1.353	±	0.128	1.365	1.208–1.498
Alike	1.261	1.155	1.215	1.210	±	0.053	1.215	1.150–1.270
Milky blue	1.176	0.994	1.077	1.082	±	0.091	1.077	0.979–1.185
Tokuso Curefast	1.144	1.137	1.015	1.099	±	0.073	1.137	1.016–1.182
Unifast III	1.389	1.307	1.377	1.358	±	0.044	1.377	1.308–1.408

**Table 3:** Comparison between groups of DLP 3D printers and self-curing resin specimens. (*n* = 3, *p* < .05 *, *p* < .01 **)

Materials		3 days	5 days
Self-curing group	DLP 3D printed group	*p*-value	p-value
Lang dental	Model	.926	.211
	Castable	.404	.853
	Clear-SG	.817	.711
	Tray	.963	.745
	Temporary	.179	.246
Alike	Model	.430	.014^*^
	Castable	.889	.308
	Clear-SG	.643	.115
	Tray	.458	.126
	Temporary	.041^*^	.018^*^
Milky blue	Model	.046^*^	.002^**^
	Castable	.286	.095
	Clear-SG	.095	.026^*^
	Tray	.051	.029^*^
	Temporary	.001^**^	.003^**^
Tokuso Curefast	Model	.046^*^	.002^**^
	Castable	.286	.095
	Clear-SG	.095	.026^*^
	Tray	.051	.029^*^
	Temporary	.001^**^	.003^**^
Unifast III	Model	.610	.179
	Castable	.151	.926
	Clear-SG	.404	.643
	Tray	.578	.676
	Temporary	.458	.211

In the 3D printing resin materials and self-curing resin materials inter-group comparison, Milky blue presented a statistically significant difference (*p* < .05, *p* < .01) in cell proliferation after 3 days compared to Model and Temporary from the inter-group comparison (Table 3). Tokuso Curefast also presented a statistically significant difference (*p* < .05, *p* < .01) in cell proliferation after 3 days compared to Model and Temporary from the inter-group comparison of cell proliferation (Table 3). Alike presented a statistically significant difference (*p* < .05) compared to Temporary from the inter-group comparison. Milky blue presented a statistically significant difference (*p* < .05, *p* < .01) compared to Tray, Clear-SG, Temporary, and Model from the inter-group comparison after 5 days (Table 3). Tokuso Curefast presented a statistically significant difference (*p* < .05, *p* < .01) compared to Tray, Clear-SG, Temporary, and Model from the inter-group comparison. Alike presented a statistically significant difference (*p* < .05) compared to Temporary and Model from the inter-group comparison.

## Discussion

In this study, the biocompatibility of dental temporary restoratives fabricated by 3D printing or by conventional self-curing methods through polishing process was compared by analyzing adhesion, morphology, and proliferation of fibroblast cells. In accordance with the experiment finding, the null hypothesis was rejected, and this study revealed that there were statistically significant differences in fibroblast cytocompatibility between 3D printing technology-based and self-curing technology-based temporary restorative resin materials.

Most previous studies on biocompatibility have conducted bacteria-related in-vitro cell experiments on temporary restorative material [24, 25]. However, only few studies have assessed the effect of temporary restorative materials fabricated through polishing process on fibroblasts.

The polishing process during the fabrication of dental restorations requires professional expertise and a deep understanding of dental materials, as this knowledge is crucial for fulfilling the functional and esthetic requirements for restoration of lost teeth according to the patient’s demands [26, 27].

In this experiment, DLP type 3D printing materials including Model, Castable, Clear-SG, Tray, and Temporary were studied. These materials were developed by Dentis, a professional dental device company in Korea. Self-curing, which is an existing temporary restorative fabrication method, was completed using Lang dental, Alike, Milky blue, Tokuso Curefast, and UniFast III. The methods of resin curing included heat-curing, self-curing, and light-curing [28]. Among these, the self-curing method is the most commonly used [29]. Excessive amount of liquid during the resin curing was reported to decrease the physical and chemical properties due to high water absorbance resulting in a decrease of the bonding strength [30, 31]. Therefore, the ratio of powder and liquid were set to be as accurate as possible by using a scale while curing the resin.

In this study, the post-treatment process was a challenging step as it may interfere with cell adhesion due to errors occurring during the experiment examining cell adhesion and morphologic patterns or due to environmental factors. SEM measurement revealed that unlike the 3D printed specimens, the self-curing specimens had a porous surface (Fig. 4). This was thought to be caused by the difference in fabrication methods between DLP 3D printing and self-curing [32, 33]. Therefore, research on biocompatibility regarding micro leakage of temporary restorative materials are recommended for future research.

Among the components examined 24 h after the leaching of the resin materials, 80–90% contained phthalate esters in plasticizer [34]. This decreases the level of the hormone estrogen and is produced after the resin curing, and may cause severe problems [35]. Despite the rareness of studies on the biocompatibility of plasticizer and remnant monomers in temporary restorative resin materials, such challenges in this study can be considered to be because of the lack of detailed information of the components of temporary restorative resin materials provided by manufacturers.

Fibroblast is the most abundant cell existing in connective tissues and controls tissue development, tissue formation, homeostasis and its maintenance [36]. Also, this is a crucial cell in diverse physiological and pathological aspects including wound healing, inflammation and cancers [37]. Fibroblast creates complex cell signaling networks through contact with other cells [38]. Fibroblast is mostly used for various cell-based therapies in a relatively easy way, as a source of cell supply. The gingiva refers to a boundary between teeth and oral mucosa, is dynamic and has the fastest rate of transformation among body tissues, being involved in immune defenses aggressively [39]. Besides, wound healing, tissue regeneration and immune function are major characteristics of the gingiva related to the therapeutic use [40]. Periodontitis, a general inflammatory disease, is widespread among adults [41]. Periodontitis is considered as permanent inflammation and tissue damage to tooth-supported structures [42]. Although this study failed to establish a mechanism of fibroblast factors that regulate genetoxicity and cytotoxicity of resin, the study result deserves to be taken note by demonstrating that biocompatibility is important when selecting dental materials. Moreover, physiological side effects of chemicals and products discharged from polymers are important issues, and further research needs to focus on this using fibroblast in the real human gingiva.

When self-curing technology is used, a chemical reaction occurs due to liquid and powder. The human body can receive harmful influence during this process. However, the latest 3D printing technology, which is designed to layer dental resin materials does not lead to a chemical reaction, compared to the existing technology. In this respect, temporary restorative resin materials printed out using the 3D printing technology are more bio-compatible with the human body, although temporary restorative resin materials manufactured using the self-curing technology and the 3D printing technology are all applicable to the human body. Besides, the 3D printing technology facilitates faster manufacture of dental prosthesis, and this makes it possible to shorten the chair time for patients at dental clinics.

Hence, it should be notable that the in-vitro cell experiment investigating biocompatibility in this study was done using specimens that are fabricated in the same manner as are the actual dental restorations, in order to model the temporary restorative resin, which is placed inside the mouth. In addition, additional experiments using fibroblasts are expected in the future with the development of 3D printing technology by studying biocompatible dental materials and complementing the limitation of the components of new materials.

## Conclusions

This study analyzed the cytocompatibility of dental materials for temporary restorations that were fabricated using DLP type 3D printing and self-curing technologies with regard to cell adhesion, morphology, and proliferation using fibroblasts involved in tissue cells and obtained the following conclusions. Increased cell adhesion and well-extended filopodia were found when using the specimens fabricated by 3D printing, and inter-group comparison showed superior biocompatibility of 3D printed specimens compared to self-curing resins. This indicates that using resins fabricated by 3D printing technology rather than the ones fabricated by self-curing technology is recommended for the fabrication of dental temporary restorations.

## Data Availability

The datasets used and/or analysed during the current study are available from the corresponding author on reasonable request.

## Abbreviations

DLP: Digital light processing
CAD / CAM: Computer aided design / computer aided manufacturing
CLSM: Confocal laser scanning microscopy
MTS: Methoxyphenyl tetrazolium salt

## References

[1] Friedman S (2002). Considerations and concepts of case selection in the management of post-treatment endodontic disease (treatment failure). Endod Topics.

[2] Angeletaki F, Gkogkos A, Papazoglou E, Kloukos D (2016). Direct versus indirect inlay/onlay composite restorations in posterior teeth. A systematic review and meta-analysis. J Dent.

[3] Yannikakis SA, Zissis AJ, Polyzois GL, Caron C (1998). Color stability of provisional resin restorative materials. J Prosthet Dent.

[4] Jensen AL, Abbott P, Salgado JC (2007). Interim and temporary restoration of teeth during endodontic treatment. Aust Dent J.

[5] Stansbury JW, Idacavage MJ (2016). 3D printing with polymers: challenges among expanding options and opportunities. Dent Mater.

[6] Güth JF, silvz JS A e, Ramberger M, Beuer F, Edelhoff D (2012). Treatment concept with CAD/CAM-fabricated high-density polymer temporary restorations. J Esthet Restor Dent.

[7] Hoshiai K, Tanaka Y, Hiranuma K (1998). Comparison of a new autocuring temporary acrylic resin with some existing products. J Prosthet Dent.

[8] Young HM, Smith CT, Morton D (2001). Comparative in vitro evaluation of two provisional restorative materials. J Prosthet Dent.

[9] Rueggeberg FA, Giannini M, Arrais CAG, Price RBT. Light curing in dentistry and clinical implications: a literature review. Braz Oral Res. 2017;31. 10.1590/1807-3107BOR-2017.vol31.0061 28902241

[10] Parandoush P, Lin D (2017). A review on additive manufacturing of polymer-fiber composites. Compos Struct.

[11] Patel DK, Sakhaei AH, Layani M, Zhang B, Ge Q, Magdassi S (2017). Highly stretchable and UV curable elastomers for digital light processing based 3D printing. Adv Mater.

[12] Osman RB, Van der Veen AJ, Huiberts D, Wismeijer D, Alharbi N (2017). 3D-printing zirconia implants; a dream or a reality? An in-vitro study evaluating the dimensional accuracy, surface topography and mechanical properties of printed zirconia implant and discs. J Mech Behav Biomed.

[13] Ngo TD, Kashani A, Imbalzano G, Nguyen KT, Hui D (2018). Additive manufacturing (3D printing): a review of materials, methods, applications and challenges. Compos Part B: Eng.

[14] Ligon SC, Liska R, Stampfl J, Gurr M, Mülhaupt R (2017). Polymers for 3D printing and customized additive manufacturing. Chem Rev.

[15] Wang X, Jiang M, Zhou Z, Gou J, Hui D (2017). 3D printing of polymer matrix composites: a review and prospective. Compos Part B: Eng..

[16] Mizrahi B (2019). Temporary restorations: the key to success. Br Dent J.

[17] Abdulmohsen B, Parker S, Braden M, Patel MP (2016). A study to investigate and compare the physicomechanical properties of experimental and commercial temporary crown and bridge materials. Dent Mater.

[18] Syed M, Chopra R, Sachdev V (2015). Allergic reactions to dental materials-a systematic review. J Clin Diagn Res.

[19] Tammaro L, Vittoria V, Calarco A, Petillo O, Riccitiello F, Peluso G (2014). Effect of layered double hydroxide intercalated with fluoride ions on the physical, biological and release properties of a dental composite resin. J Dent.

[20] Borelli B, Zarone F, Rivieccio V, Riccitiello F, Simeone M, Sorrentino R, Procino A (2017). Polyacrylic resins regulate transcriptional control of interleukin-6, gp80, and gp130 genes in human gingival fibroblasts. J Oral Sci.

[21] Figueredo CM, Martins AP, Lira-Junior R, Menegat JB, Carvalho AT, Fischer RG, Gustafsson A (2017). Activity of inflammatory bowel disease influences the expression of cytokines in gingival tissue. Cytokine.

[22] Kostic M, Pejcic A, Igic M, Gligorijevic N (2017). Adverse reactions to denture resin materials. Eur Rev Med Pharmacol Sci.

[23] Antohe ME, Dascalu C, Savin C, Forna NC, Balan A (2016). Study regarding the toxic effects of acrylic resins. Mat Plast.

[24] Adnan S, Khan FR (2016). Comparison of micro-leakage around temporary restorative materials placed in complex endodontic access cavities: an in-vitro study. J Coll Physicians Surg Pak.

[25] Moradi S, Lomee M, Gharechahi M (2015). Comparison of fluid filtration and bacterial leakage techniques for evaluation of microleakage in endodontics. Dent Res J.

[26] Awad D, Stawarczyk B, Liebermann A, Ilie N (2015). Translucency of esthetic dental restorative CAD/CAM materials and composite resins with respect to thickness and surface roughness. J Prosthet Dent.

[27] Rekow D, Nappi B, Zhu Y. Method and apparatus for modeling a dental prosthesis. U.S. Patent No. 5,273,429. 1993.

[28] Chen R, Han Z, Huang Z, Karki J, Wang C, Zhu B, Zhang X (2017). Antibacterial activity, cytotoxicity and mechanical behavior of nano-enhanced denture base resin with different kinds of inorganic antibacterial agents. Denl Mater J.

[29] Ferracane JL, Stansbury J, Burke FJT (2011). Self-adhesive resin cements–chemistry, properties and clinical considerations. J Oral Rehabil.

[30] Palitsch A, Hannig M, Ferger P, Balkenhol M (2012). Bonding of acrylic denture teeth to MMA/PMMA and light-curing denture base materials: the role of conditioning liquids. J Dent.

[31] Wang X, Huyang G, Palagummi SV (2018). High performance dental resin composites with hydrolytically stable monomers. Dent Mater.

[32] Dikova TD, Dzhendov DA, Ivanov I, Bliznakova K (2018). Dimensional accuracy and surface roughness of polymeric dental bridges produced by different 3D printing processes. Arch Mater Sci Eng.

[33] Hou K, Ai T, Liu R, Xiang N, Jin J, Hu W, Luo B. Modeling chronic Dacryocystitis in rabbits by nasolacrimal duct obstruction with self-curing resin. J Ophthalmol. 2017. 10.1155/2017/3438041 PMC5498895 28717520

[34] Gautam R, Singh RD, Sharma VP, Siddhartha R, Chand P, Kumar R (2012). Biocompatibility of polymethylmethacrylate resins used in dentistry. J Biomed Mater Res B: Applied Biomaterials.

[35] Soh NHBC, Pandian S (2019). Reactions to acrylic resin in orthodontic patient. Res J Pharm Techno.

[36] Räsänen K, Vaheri A (2010). Activation of fibroblasts in cancer stroma. Exp Cell Res.

[37] Häkkinen L, Larjava H, Fournier BP (2014). Distinct phenotype and therapeutic potential of gingival fibroblasts. Cytotherapy.

[38] Fournier BP, Larjava H, Häkkinen L (2013). Gingiva as a source of stem cells with therapeutic potential. Stem Cells Dev.

[39] Abraham S, Deepak KT, Ambili R, Preeja C, Archana V (2014). Gingival biotype and its c linical significance–a review. Saudi J Dent Res.

[40] Soto-Barreras U, Cortés-Sandoval G, Dominguez-Perez R, Loyola-Leyva A, Martinez-Rodriguez PR, Loyola-Rodriguez JP (2017). Stimulatory effect of Aggregatibacter actinomycetemcomitans DNA on proinflammatory cytokine expression by human gingival fibroblasts. Arch Oral Biol.

[41] Moutsopoulos NM, Kling HM, Angelov N, Jin W, Palmer RJ, Nares S, Wahl SM (2012). Porphyromonas gingivalis promotes Th17 inducing pathways in chronic periodontitis. J Autoimmun.

[42] Haghnegahdar A, Khosrovpanah H, Andisheh-Tadbir A, Mortazavi G, Moghadam MS, Mortazavi SMJ, Koohi O (2014). Design and fabrication of helmholtz coils to study the effects of pulsed electromagnetic fields on the healing process in periodontitis: preliminary animal results. J Biomed Phys Eng.

